# Umbilical cord mesenchymal stem cells derived extracellular vesicles can safely ameliorate the progression of chronic kidney diseases

**DOI:** 10.1186/s40824-016-0068-0

**Published:** 2016-08-05

**Authors:** Wael Nassar, Mervat El-Ansary, Dina Sabry, Mostafa A. Mostafa, Tarek Fayad, Esam Kotb, Mahmoud Temraz, Abdel-Naser Saad, Wael Essa, Heba Adel

**Affiliations:** 1 Department of Internal Medicine, Nephrology Section, Sahel Teaching Hospital, General Organization of Teaching Hospitals and Institutes (GOTHI), Cairo, Egypt; 2 Department of Internal medicine, Nephrology Section, Faculty of Medicine, October Six University, Cairo, Egypt; 3 Department of clinical pathology, stem cells Section, Faculty of medicine, Cairo University, Cairo, Egypt; 4 Department of Internal Medicine, Nephrology Section, Faculty of medicine, Cairo University, Cairo, Egypt; 5 Department of Biochemistry, Faculty of medicine, Cairo University, Cairo, Egypt

**Keywords:** Chronic kidney disease, Extracellular vesicles, Mesenchymal stem cells, Microvesicles

## Abstract

**Background:**

Bio-products from stem/progenitor cells, such as extracellular vesicles, are likely a new promising approach for reprogramming resident cells in both acute and chronic kidney disease. Forty CKD patients stage III and IV (eGFR 15–60 mg/ml) have been divided into two groups; twenty patients as treatment group “A” and twenty patients as a matching placebo group “B”. Two doses of MSC-derived extracellular vesicles had been administered to patients of group “A”. Blood urea, serum creatinine, urinary albumin creatinine ratio (UACR) and estimated glomerular filtration rate (eGFR) have been used to assess kidney functions and TNF-α, TGF-β1 and IL-10 have been used to assess the amelioration of the inflammatory immune activity.

**Results:**

Participants in group A exhibited significant improvement of eGFR, serum creatinine level, blood urea and UACR. Patients of the treatment group “A” also exhibited significant increase in plasma levels of TGF-β1, and IL-10 and significant decrease in plasma levels of TNF-α. Participants of the control group B did not show significant improvement in any of the previously mentioned parameters at any time point of the study period.

**Conclusion:**

Administration of cell-free cord-blood mesenchymal stem cells derived extracellular vesicles (CF-CB-MSCs-EVs) is safe and can ameliorate the inflammatory immune reaction and improve the overall kidney function in grade III-IV CKD patients.

## Background

Mesenchymal stromal cells (MSCs) could reverse acute and chronic kidney injury [1]. The majority of the transplanted MSCs are trapped by the liver, lung and spleen whereas only less than 1 % is localized at targeted tissue supporting the notion that the efficacy of MSCs in treating diseases is independent of engraftment and differentiation [2]. Extracellular vesicles (EVs) derived from MSCs have recently exploited in regenerative medicine to repair damaged tissues. These membranous structures deliver bioactive molecules, such as proteins, mRNAs, micro-RNAs, bioactive lipids and signaling receptors that can horizontally transfer genetic information [3–5]. It has also been reported that MSCs-EVs can protect AKI in ischemia reperfusion injury (IRI) in animal models [6]. In addition to multipotent capabilities of MSC-derived EVs, it has been shown to modulate both innate and adaptive immune responses and mediate immune suppressive effects through suppression of T-cell proliferation and enhancement of proliferation of the regulatory T lymphocytes (CD4-CD25-FOXP3 T-reg.) [7, 8]. Few studies addressed the potential benefit of MSC in treatment of chronic kidney disease (CKD) [9, 10]. The effect of MSCs-EVs both in the acute and chronic models was attributed to their paracrine action rather than a trans-differentiation of renal resident cells [11–13]. In this context, MSCs-EVs may act by mitigating injury and/or favoring repair to cells which survived the injury. Recently, Food and Drug Administration has licensed biologic applications for minimally manipulated, unrelated allogeneic placental/umbilical cord blood intended for hematopoietic and immunological reconstitution in patients with disorders affecting the hematopoietic system [14].

Repeated rather than a single injection of MSC-EVs prevents renal fibrosis from occurring after ischemia-reperfusion injury (IRI) [15, 16]. MSCs-EVs also exert potent anti-inflammatory and anti-fibrotic effects and may therefore indirectly improve renal function by reducing disease associated inflammation and fibrosis. Thus, MSC-EVs provide a new effective therapeutic approach to slow the progression of CKD and improve renal function. MSCs-EVs appear to be an alternative therapeutic approach to MSCs that qualifies them as a very promising tool for future regenerative and immune modulating therapies. Moreover, the potential risks associated with stem cell therapy, such as mal-differentiation or tumor development, could be avoided. Besides, cryopreserved EVs can be injected directly and repeatedly thus can be a ready-to-use off-shelf drug [16].

This study was conducted to assess the safety and therapeutic efficacy of cell-free human cord blood derived extracellular vesicles in ameliorating the progression of grade III & IV chronic kidney disease (eGFR 15–60 mg/ml) patients based on the anti-inflammatory, anti-fibrotic and anti-apoptotic properties of MSC-EVs regardless the etiology of chronic kidney disease.

## Patients and methods

In a single-center, randomized, placebo-controlled, phase II/III clinical pilot study, forty CKD patients who have been diagnosed for more than 6 months, age between 26–44 years (Mean age 32.154 ± 9.21 years) with eGFR between 15–60 mg/ml/min. The patients had been divided into two groups; group A, twenty CKD patients (Table 1) treated by two doses of MSC-EVs, intra-arterial and intravenous injections and a control group B (Table 2) of another twenty CKD patient as a matching placebo group. The control group patients had been given only intravenous saline (sham therapy) but no intra-arterial injections were administrated.

**Table 1:** Entry selection criteria of the treatment group (A) patients

Patient	Age	Gender	CKD	Weight	Comorbidities	UACR	S. Creat.	Bl. Urea	e-GFR
No/G	Yrs		years	Kgm.		mg/g	mg/dl	mg/dl	ml/min.
1/A	40	M	6	77	DM, HTN	1135	3.1	122	30
2/A	26	F	8	64	DM, SLE, HTN	1527	4.3	124	32
3/A	35	F	5	62	IN, HTN	1542	3.5	125	32
4/A	44	F	7	78	IN, HTN	1375	3.6	131	37
5/A	26	M	9	63	HTN	1298	3.8	124	26
6/A	37	M	8	65	DM, HTN	1273	3.1	122	29
7/A	33	F	7	61	DM, IN	1722	4.1	143	26
8/A	34	F	12	62	IN, HTN	1372	2.4	112	31
9/A	28	F	7	69	SLE, HTN	1578	4.1	152	28
10/A	30	F	8	60	HTN	1677	3.3	127	41
11/A	29	F	9	62	SLE, HTN	1744	3.2	124	29
12/A	40	F	13	59	DM	2133	2.8	113	34
13/A	37	M	7	69	DM, HTN	1673	3.6	132	32
14/A	35	M	15	61	IN, HTN	1273	3.8	148	37
15/A	39	M	9	68	DM	1422	3.2	121	32
16/A	41	M	6	59	DM, HTN	1566	2.6	117	34
17/A	37	M	8	74	DM	1433	3.7	134	26
18/A	28	F	12	63	HTN	2133	2.9	116	29
19/A	34	F	9	68	DM, IN	1573	3.8	87	25
20/A	28	M	11	77	HTN	1677	3.8	132	25

Mean age 32.154 ± 9.21year. 50% were males. The median body weight 66.05 ± 9.31 kgm. Seventy five percent (15/20) were hypertensive, fifty percent (10/20) were T1D, twenty five percent (5/20) were interstitial nephritis and fifteen percent (3/20) were systemic lupus erythematosus. (IN, interstitial nephritis; S. creat. Serum creatinine; DM, diabetes mellitus; HTN, hypertension; eGFR, estimated glomerular filtration rate)

**Table 2:** Entry selection criteria of the control group (B) patients

Patient	Age	Gender	CKD	Weight	Comorbidities	UACR	S. Creat.	Bl. Urea	e-GFR
No/G	Yrs		years	Kgm.		mg/g	mg/dl	mg/dl	ml/min.
1/B	38	M	7	72	IN, HTN	1266	3.5	112	36
2/B	36	F	11	67	DM, SLE, HTN	1427	3.3	114	34
3/B	32	M	6	65	DM, HTN	1562	3.2	123	31
4/B	40	F	5	68	IN, HTN	1673	3.7	121	32
5/B	28	F	7	73	DM, SLE	1458	3.1	134	41
6/B	36	M	6	66	IN, HTN	1403	3.5	132	32
7/B	37	F	6	67	HTN	1643	3.1	123	36
8/B	33	F	9	72	IN, HTN	1474	3.4	122	34
9/B	32	F	11	64	SLE, DM	1372	3.1	132	38
10/B	29	M	6	67	SLE, HTN	1575	3.3	147	31
11/B	31	F	8	62	HTN	1644	3.5	114	34
12/B	37	F	9	69	DM, HTN	1733	3.8	123	24
13/B	34	M	7	64	DM	1376	2.6	125	39
14/B	37	M	12	65	DM, HTN	1773	3.4	124	38
15/B	38	F	11	66	HTN	1352	3.6	117	39
16/B	28	M	9	69	SLE, HTN	1467	3.6	126	36
17/B	41	M	5	64	DM	1463	2.7	134	36
18/B	32	M	7	73	SLE, HTN	1423	2.7	126	39
19/B	37	F	8	65	DM, IN	1378	3.5	113	35
20/B	29	M	8	73	HTN	1748	3.3	98	29

Mean age 34.22 ± 6.21 year. 50 % were males. The median body weight 64.15 ± 7.31 kgm. Seventy percent (14/20) were hypertensive, forty five percent (9/20) were T1D, twenty five percent (5/20) were interstitial nephritis and thirty percent (6/20) were systemic lupus erythematosus. (IN, interstitial nephritis; S. creat. Serum creatinine; DM, diabetes mellitus; HTN, hypertension; eGFR, estimated glomerular filtration rate)

Entry selection criteria included normal liver functions and absence of chronic or recurrent infection for the last 12 months. The primary endpoint was the safety of the therapy all through the study period of one year; and preliminary evaluation of the possible adverse effects (e.g. allergic reactions, anaphylaxis and opportunistic infections) that might take place. The secondary endpoint was the efficacy of treatment assessed by duplication of eGFR or fifty percent reduction of serum creatinine from the baseline of each patient. Using serum creatinine, the Bedside Schwartz equation was used to estimate the glomerular filtration rate (eGFR).

Recently, Oscar and collaborators observed a positive dose-dependent response of the total tissue fluorescence following IV injection of 3 different doses: 0.25x1010, 1.0x1010 and 1.5x1010 p/g of DiR-labelled EVs. EVs accumulated mainly in liver, spleen, gastrointestinal tract and lungs. The degree of saturation of the mononuclear phagocyte system (also known as the reticuloendothelial system, RES) may explain EVs accumulation in these organs. The intermediate dose (1x1010 p/g) was determined to be sufficient for detection in all examined organs with minimal saturation [17]. This was the dose that we used in our experiments. The route of injection influenced tissue distribution of infused EVs and the site of injection alternations may thus be used to increase the EV distribution to a potential tissue target [17]. Due to the very small size of EVs (20–1000 nm) we had no fear of size related complications (i.e. engraftment syndrome associates MSCs transplantation) and intra-arterial EVs injection has been successfully used to reduce myocardial ischemia/reperfusion injury [18]. So, we’ve chosen the intra-arterial rout to maximize EVs delivery to the kidneys.

**Fig. 1:**
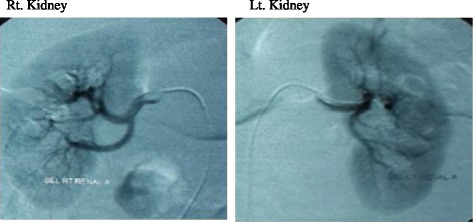
Computerized Tomographic scanning during injection of MSCs EVs (Rt. & Lt. kidneys)

The first dose was an intravenous injection through the median cubital vein to patients of group A with one week apart, a second dose was selective (Rt. & Lt.) intra-renal arteries C-T guided administration (Fig. 1) of cell free cord-blood mesenchymal stem cells extracellular vesicles (CF-CB-MSCs-EVs) at 100 μg/kg/dose (one cord for each patient). Each cord (25–30 cm long) produces about 120–150 × 106 MSCs and each one million MSCs produces 80–150 μg of proteins [19]. To quantify the protein content, the Bradford protein assay kit (P0006, of Beyotime Institute of Biotechnology, China) was used in the Bradford assay.

Some of the patients of those who achieved the target of the study which is duplication of eGFR or decrease of serum creatinine by more than 50 % from the study entrance underwent kidney biopsy for thorough histopathologic assessment.

### Ethical aspects

The food and drug administration (FDA) in the USA has released a biologic license for applications of minimally manipulated, unrelated allogeneic placental/umbilical cord blood intended for hematopoietic and immunologic reconstitution in patients with disorders affecting the hematopoietic system in 2014 [14]. The study protocol has been approved by the health ethical committee of Sahel Teaching Hospital on April 2014. All patients have been informed verbally and a written consent has been signed prior any step according to Amsterdam’s declarations. A written consent from both parents (for umbilical cord donors) has been obtained prior to any intervention.

### Isolation and characterization of MSCs

The MSCs were cultured in the presence of Mesenchymal Stem Cells Basal Medium (MSCBM, Lonza). To expand the MSCs, the adherent monolayer was detached by trypsin treatment for 5 min at 37C, after 3 days for the first passage and every 2 days for subsequent passages (with a maximum of six passages). At each passage, cells were counted and analyzed for immune-phenotype by flow-cytometric analysis. MSCs expressed -CD44, −CD73, −CD34, −CD45, CD80, −CD86 and did not express hematopoietic markers like CD45, CD14 and CD34. They also did not express the co-stimulatory molecules (CD80, CD86 and CD40) [20].

### Isolation and characterization of MSCs-EVs

MSCs were obtained from supernatants of human cord blood mesenchymal stem cells (hCB-MSCs) as previously described [19]. Briefly, hCB-MSCs were cultured in DMEM without FBS and with added 0.5 % human serum albumin (HSA) (Sigma-Aldrich) overnight. The viability of the cell cultured overnight was > 99 % as detected by trypan blue exclusion and no apoptotic cells were detected by terminal transferase-mediated dUTP nick-end labeling (TUNEL) assay. The conditioned medium was collected and stored at −80 °C. The medium was centrifuged at 2,000 g for 20 min to remove debris, and then ultracentrifuged at 100,000 g in a SW41 swing rotor (Beckman Coulter, Fullerton, CA, USA) for one hour at 4 °C. EVs were washed once with serum free M199 (Sigma-Aldrich) containing 25 mM HEPES (pH = 7.4) and submitted to a second ultracentrifugation in the same conditions. EVs were stored at −80 °C for the experiments. To quantify the protein content, the Bradford assay kit (P0006, Beyotime Institute of Biotechnology, China) was used in the Bradford assay.

**Fig. 2:**
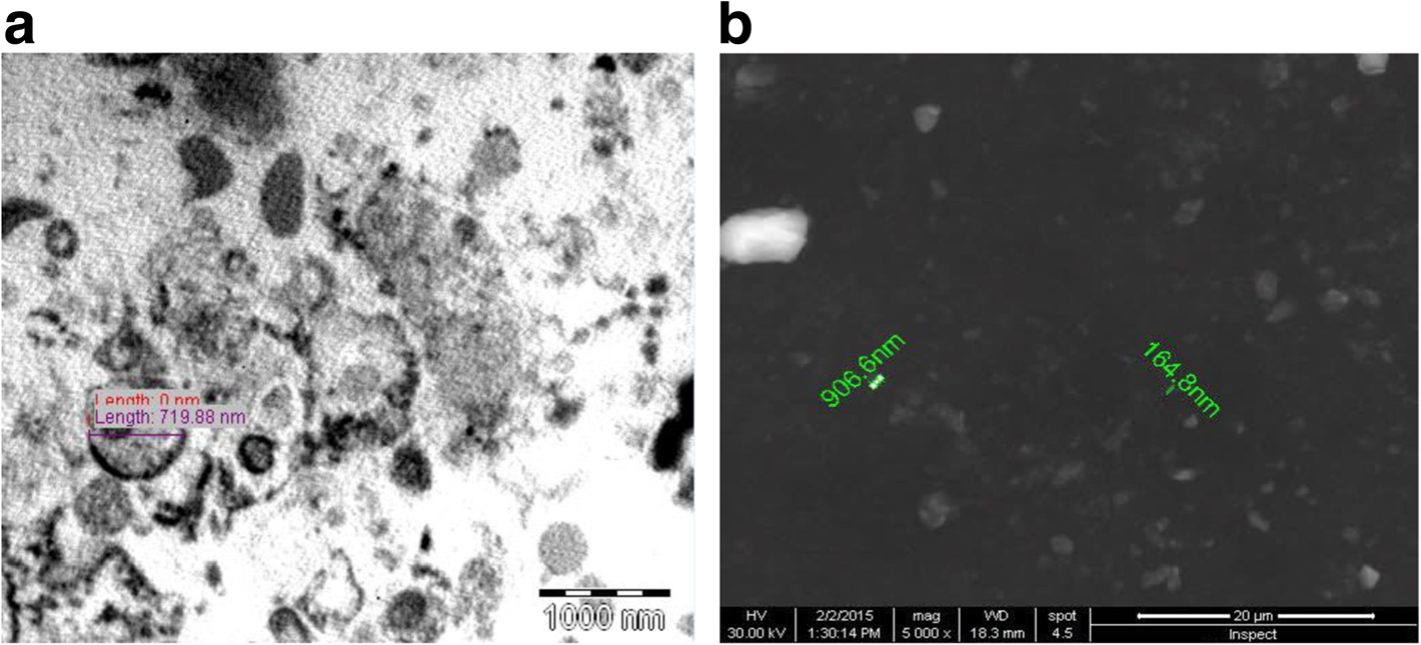
Electron micrographs of mesenchymal stem cell-derived vesicles isolated by differential centrifugation. **a** before filtration (**b**) after filteration, (exosomes, diameter <100 nm), (microvesicles, diameter 100–1000 nm). Please notice the larger size of apoptotic vesicles compared with the other types of vesicles

EVs were fixed with 2.5 % glutaraldehyde in HSA for 2 h, after being washed; EVs were ultra-centrifuged and suspended in 100 uL HSA. A total of 20 uL of EVs was loaded onto a formvar/carbon-coated grid, negatively stained with 3 % aqueous phosphor-tungstic acid for one minute and observed by transmission electron microscopy (HITACHI, H-7650, Japan). Transmission and scanning electron microscopy (Fig. 2) performed on purified EVs showed their spheroid morphology and confirmed their size [10].

Flow cytometry analyses EVs were detected mainly below the forward scatter signal corresponding to 1- mm beads. By Zetasizer Nano (Malvern Instruments, Malvern Worcestershire, United Kingdom), that is an instrument that permit to discriminate micro-particles inferior to 1 mm of diameter, the size of EVs ranged from 80 nm to 1000 nm, with a mean value of 435 nm. Flow cytometry was used to characterize the isolated EVs. EVs were incubated for 30 min at room temperature, 5 ml of latex beads were added and incubated for another 30 min at 4 °C, then washed in 0.5 % BSA in PBS and incubated with different antibodies (CD9, CD45, CD63, CD73 or with appropriate isotype control IgG. After washing, EV-coated beads were immediately analyzed using a FACS Calibur flow cytometer (Becton Dickinson, FACS Calibur). Flow cytometry analyses showed the presence of several adhesion molecules known to be expressed on MSC plasma membrane such as CD44, CD29, α4- and α5 integrins and CD73, but not α6-integrin [21]. In addition, EVs did not express HLA-class I at variance with the cells of origin or HLA-class II. The morphological analyses performed on EVs suspension after staining with propidium iodide did not show the presence of apoptotic bodies [10].

### Kidney biopsy

Tru-cut needle renal biopsy was done in three responders. We assessed tubular cell proliferation after MSCs EVs treatment using rabbit anti-Ki67 mAbs. Antibodies to CD133, which marks putative human kidney progenitors, were used to ascertain whether CD133 is also up-regulated by injury in differentiated proximal tubules.

### Statistical analysis

Statistical analysis was performed by using the t-tests, analysis of covariance (ANCOVA) tests as appropriate. A Covariance *P*-value of < 0.05 was considered significant. All patients were included in the safety analyses. The primary efficacy end point was the change in eGFR and serum creatinine between baseline and follow-up.

## Results

### Safety of cell-free cord-blood MSCs derived microvesicles therapy

Forty CKD patients were enrolled in this study. Median age was 24.65 ± 4.705 years (range: 19 to 34), and median CKD duration was 3.6 ± 0.754 years (range: 2 to 5). Participants were randomly assigned to receive cell-free cord blood MSCs derived extracellular vesicles (*n* = 20) or sham therapy as control group B (*n* = 20). Each participant in group “A” received two doses with one week apart, the first dose was intravenous and the second was intra-arterial.

No participants experienced any significant adverse events during or after treatment and throughout the study period (one year). Most of the patients experienced mild discomfort during puncture of the femoral artery, but discomfort resolved quickly following the conclusion of the procedure.

#### Efficacy outcomes in improving kidney functions (Figs. 3, 4, 5, 6)

**Fig. 3:**
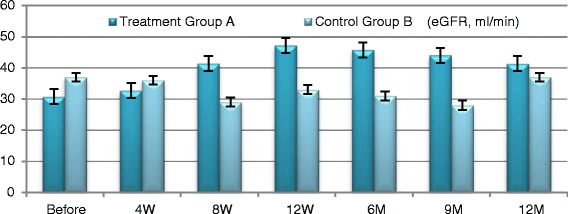
eGFR changes in both groups

**Fig. 4:**
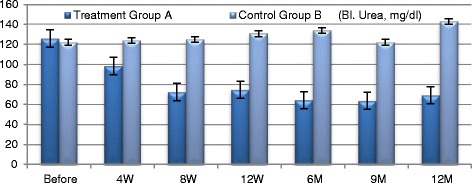
Blood urea changes in both groups

**Fig. 5:**
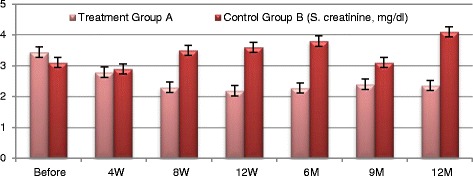
Serum creatinine changes in both groups

**Fig. 6:**
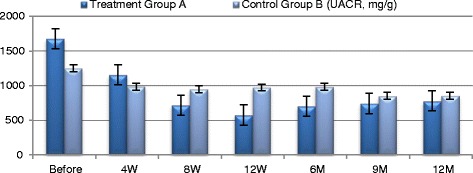
UACR changes in both groups

Participants in Group A exhibited improved **eGFR** from baseline of (31.05 ± 4.513) ml/min to (47.25 ± 5.139) ml/min. at 12 week (*P* ≤ 0.0094) and to (41.35 ± 2.587) ml/min. at the end of one year which was not statistically significant. **Blood urea** has been declined from baseline of (125.8 ± 14.16) mg/dl to (74.65 ± 6.907) mg/dl. At 12 weeks and to (69.2 ± 2.977) mg/dl by the end of the year which were both of no statistical significance. **Serum creatinine** decreased from baseline of (3.435 ± 0.493) mg/dl to (2.185 ± 0.252) mg/dl at 12 weeks (*P* ≤ 0.044) and to (2.355 ± 0.211) by the end of the year which was not statistically significant. Urinary albumin creatinine ratio **(UACR)** has improved from baseline of (1555 ± 259.3) mg/g to (574.3 ± 57.6) mg/g at 12 weeks and to (788.65 ± 89.84) mg/g by the end of one year; which were both of no statistical significance. Participants in the control Group B did not exhibit significant changes of any of these aforementioned parameters during the follow-up period.

#### Efficacy outcomes in immune regulation

Next, we explored mechanisms underlying MSCs extracellular vesicles mediated immune modulation. We measured changes in TGF-β1 which has been implicated in Treg-mediated immune suppression [20] as well as in maintenance of self-tolerance in animal models subjected to stem cell-mediated immune modulation. IL-10 is a cytokine with multiple pleiotropic effects in immune-regulation and inflammation. IL-10 can block NF-кB activity, and is involved in the regulation of the Jak-Stat signaling pathway. Blocking the Jak-Stat signaling pathway may provide an effective means for preventing immune response via immunological and metabolic effects [22]. We examined TGF-β1, IL-10 and TNF-α expression to explore whether these pathways are activated following cell-free CB-MSCs microvesicles therapy.

**Fig. 7:**
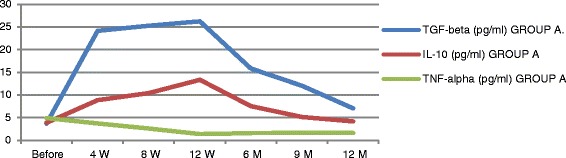
Markers of immune modulation throughout the study period

**Fig. 8:**
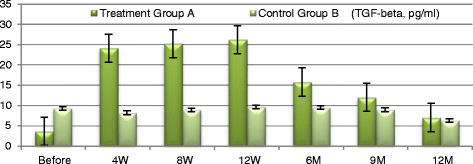
TGF-β changes in both groups

**Fig. 9:**
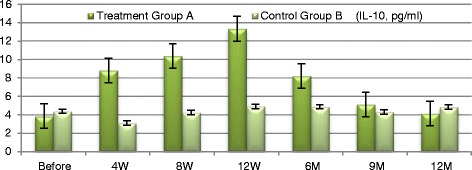
IL-10 changes in both groups

**Fig. 10:**
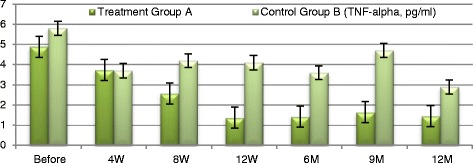
TNF-α changes in both groups

At baseline participants of the treatment group A exhibited significant increases in plasma level of TGF-β1 from baseline of (3.682 ± 0.546) to (26.23 ± 4.34) at 12 weeks (*P* ≤ 0.0035) and to (7.02 ± 2.001) at the end of the study period. Group A patients exhibited significant increase in plasma levels of IL-10 from baseline (3.8535 ± 0.662) to (13.33 ± 1.516) at 12 weeks (*P* ≤ 0.01) and to (4.152 ± 0.669) at the end of the study period (*P* = 0.084). TNF-α has been significantly decreased in the treatment group A from baseline of (4.98 ± 1.466) to (1.635 ± 0.231) at 12 weeks (*P* ≤ 0.005) and to (1.45 ± 0.148) at the end of the study period (*P* < 0.009) with persistent significant decrease of TNF-α levels (Figs. **7**, 8, 9 and 10). TGF-β1, IL-10 and TNF-α have all failed to show significant changes in the control group B.

#### Immuno-histo-pathology

**Fig. 11:**
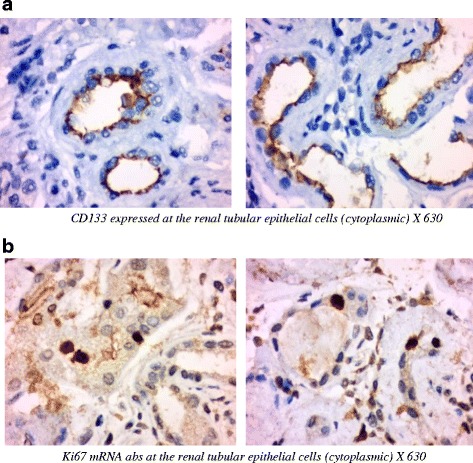
Kidney biopsy of a patient of the treatment group (HTN nephrosclerosis) x 630: Panel (**a**) showing the expression of CD133 in the renal tubular epithelial cells indicating active recovery of renal tubular cells. Panel (**b**) showing the expression of Ki67 mRNA abs indicating active proliferation of tubular epithelial cells. Both markers had not been shown in a similarly stained kidney biopsy of the control group. This represents strong evidence that these changes are related to the MSC-EVs

**Fig. 12:**
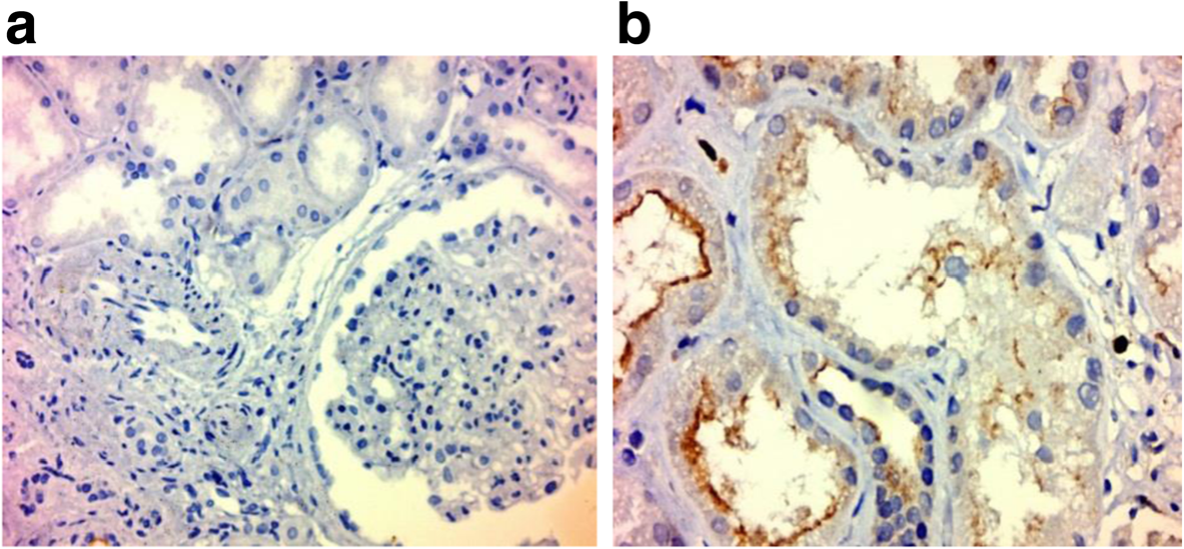
Kidney biopsy pictures of a patient of the control group (HTN nephrosclerosis) x 100; for CD133 (Panel **a**) and Ki67 (Panel **b**). CD133: no tubular cytoplasmic staining was detected panel (**a**). Ki67 mRNA abs: very few and scattered positive tubular cells and few inflammatory cells within peritubular capillaries panel (**b**) compared to the study group. Note that low power was to widen the examination field

We assessed tubular cell proliferation after MSCs EVs treatment using rabbit anti-Ki67 mAbs, we assessed the ability of differentiation of tubular cell in response to kidney injury using anti CD133 mAbs. Tubular cells mRNAs encoding CD133 are upregulated in differentiated epithelia after injury. Our results show linear expression of CD133 and Ki67 in both tubular epithelial cells and within the inflammatory cells which are a good marker of proliferation and/or dedifferentiation of the tubular cells (Fig. 11). Kidney biopsy of a patient of the controls (Fig. 12) did not show tubular cytoplasmic staining of CD133. Very few and scattered positive tubular cells and few inflammatory cells within peritubular capillaries of Ki67 mRNA abs compared to the study group*.*

## Discussion

MSCs are known to exert potent anti-inflammatory, anti-apoptotic and anti-fibrotic effects and may therefore indirectly improve renal functions by reducing disease associated inflammation and fibrosis through their paracrine effects [23]. Thus, MSC-EVs therapy may be an effective new approach to slow the progression of CKD and improve renal functions [10]. Recently, several studies demonstrated that the administration of MSCs reverses AKI in different experimental models [8, 24, 25]. The mechanisms of kidney repair have been mainly ascribed to a paracrine support of MSC. Administration of conditioned medium from MSCs may mimic the beneficial effects of the MSC administration, indicating that the tubular engraftment of the MSCs is not necessary [19]. The present study demonstrates the safety and efficacy of Cell-Free Cord-blood MSCs derived extracellular vesicles (CF-CB-MSCs-EVs) in improving the immune inflammatory process in CKD patients regardless the etiology of the chronic kidney disease. Our data are consistent with these findings as our patient showed significant, though transient, improvement in their kidney functions.

Intravenously administration of MSC-EVs, has the same efficacy of MSCs on the functional and morphological recovery of glycerol-induced AKI in SCID mice [19]. Single administration of EVs ameliorates renal function and morphology and improves survival. However, survived mice showed chronic tubular injury and persistent increase in BUN and creatinine. Administration of multiple injections of MSC-EVs, further decrease the mortality and survival with normal histology and renal function [26].

While the intravenous (IV) rout can exert an anti-inflammatory effect to the circulating lymphocytes as evident by the increase of TGF-β1 and IL-10 levels; the intra-arterial (IA) rout may exert better means to deliver effective amount of EVs to renal tubular cells to facilitate horizontal transfer of EVs cargo of miRNA and mRNA to tubular epithelial cells. Intravenous EVs may educate the circulating immune cells by their anti-inflammatory effect. While intra-arterial EVs administration may modulate local environment, activate endogenous progenitor cells to force the tubular epithelial cells to admit and accommodate the EVs cargo and facilitate cell recovery.

Clinical improvement has been parallel to modulation of the immune inflammatory markers of those patients as evident by significant rise of TGF-β1 and IL-10 levels and significant decrease of TNF-α levels in response to treatment. It is known that TGF-β1 is a key regulator of the production and re-modeling of the extracellular matrix through its immune-modulatory mechanism by suppressing the effector T-cell proliferation and enhancing the proliferation of the regulatory CD4 CD25 FOXP3 T-cell population [26, 27]. This effect can be differentiation [28] and/or dedifferentiation [28] and/or re-arrangement of stromal cells. Besides, IL-10 is a cytokine with multiple pleiotropic effects in immune-regulation and inflammation. IL-10 can block NF-кB activity, which is involved in the regulation of the Jak-Stat signaling pathway. Blocking the Jak-Stat signaling pathway may provide an effective means for preventing immune activity via immunological and metabolic effects [22]. MSCs derived extracellular vesicles might exhibit immune-modulatory effect through specific and nonspecific anti-inflammatory properties [5]. Moreover, Tumor necrosis factor (TNF-α, cachexin, or cachectin) is a cell signaling protein involved in systemic inflammation and is one of the cytokines that make up the acute phase reaction that may frequently exacerbate during the course of chronic illnesses [25, 29]. Tumor necrosis factor is also involved in NF-кB activation as MAPK pathways and induction of death signaling and is generally a pro-apoptotic factor. The myriad effects mediated by these pathways indicate the existence of extensive cross-talk between different cytokines and reactive oxygen species in response to tumor necrosis factor (TNF-α) release [18]. In this context, various cells with vastly diverse functions and conditions can all respond appropriately to inflammation. Thereby, the significant increase of TGF-β1 and IL-10 levels as well as significant decrease in TNF-α levels following MSCs derived EVs treatment are strong evidences of regression of inflammation and apoptosis which have a pivot role in development and progression of the pathogenesis of CKD. Modulation of these immune parameters can explain the significant transient improvement in clinical parameters of the treated patients.

Though kidney biopsy after 3 months in some of the responding patients did not show significant histologic changes; however, using rabbit anti-Ki67 mAbs antibodies showed expression of Ki67(a marker of regeneration) in some renal tubular cells confirming the ability of MSC-EVs to activate renal tubular cells to regenerate. Tubular cells mRNAs encoding CD133 has been also up regulated in differentiated epithelia after injury (Fig. 12). Previous work has shown that CD133 encoded cells have special qualities such as clonal expansion, sphere formation, and the ability to ameliorate injury [30]. Our results show linear expression of CD133 and scattered Ki67 expression in the tubular epithelial cells which is a good indicator of recovery and active proliferation and/or dedifferentiation of renal tubular cells.

Interestingly, the improvement pattern among group “A” patients was heterogeneous, as some patients exhibited improvement few days after the first dose whereas, others showed improvement many days after the second dose. Consistent with this observation; S. Bruno et al., [25], reported the superior effect of multiple rather than single injection of EVs in improvement of renal functions in CKD. Besides, this heterogeneity can be attributed to the polyclonal nature of the pathogenesis of CKD and hence the response to the therapeutic effect of the EVs due to interference with cytokines in immune intervention which is a complex matter depending on the timing, dose, and route of administration etc.. However, longer observations and larger patient’s samples are needed to confirm these results.

## Conclusion

Administration of two doses, first intravenous and second intra-arterial targeting the diseased organ, of cell free cord-blood mesenchymal stem cells extracellular vesicles (CF-CB-MSCs-EVs) with one week apart is safe and can ameliorate the inflammatory immune reaction and transiently (3–6 months) improve the overall kidney function in grade III-IV CKD patients. Administration of MSCs derived EVs mimic the beneficial effects of the MSC administration. Besides, the potential risks associated with stem cell therapy, such as mal-differentiation or tumor development could be avoided. Moreover, cryopreserved EVs can be injected directly and repeatedly thus appearing to be a ready-to-use drug. Bio-products from stem/progenitor cells, such as extracellular vesicles and their contents, are likely a new promising approach for reprogramming resident cells in chronic kidney diseases. This approach can also be applied to a wide variety of autoimmune diseases as a means of therapy of diseased cells (i.e. reprogramming) and not cell therapy.

## Declarations

The study protocol has been approved by the health ethical committee of Sahel Teaching Hospital on April 2014. All patients have been informed verbally and a written consent for the research and publication has been signed prior any step according to Amsterdam’s declarations. No individual patient’s data were included. A written consent from both parents (for umbilical cord donors) has been obtained prior to any intervention.

## Abbreviations

ANCOVA, Analysis of Covariance; BUN, Blood Urea Nitrogen; CF-CB-MSCs-EVs, Cell-free cord-blood mesenchymal stem cells derived extracellular vesicles; CKD, Chronic Kidney Disease; eGFR, Estimated Glomerular Filtration Rate; EVs, Extracellular Vesicles; HGT, Horizontal Gene Transfer; IA, Intra-arterial; IL, Interleukin; IV, Intravenous; MAPK, Mitogen-activated protein kinases; MSCs, Mesenchymal Stem Cells; NF-кB, Nuclear Factor Kappa Beta; SCID, severe combined immunodeficiency; TGF-β, Transforming Growth Factor Beta; TNF-α, Tumor Necrosis Factor alpha; UACR, Urinary Albumin Creatinine Ratio
