# Erratum to: Umbilical cord mesenchymal stem cells derived extracellular vesicles can safely ameliorate the progression of chronic kidney diseases

**DOI:** 10.1186/s40824-017-0089-3

**Published:** 2017-04-06

**Authors:** Wael Nassar, Mervat El-Ansary, Dina Sabry, Mostafa A. Mostafa, Tarek Fayad, Esam Kotb, Mahmoud Temraz, Abdel-Naser Saad, Wael Essa, Heba Adel

**Affiliations:** 1 Department of Internal Medicine, Nephrology Section, Sahel Teaching Hospital, General Organization of Teaching Hospitals and Institutes (GOTHI), Cairo, Egypt; 2 Department of Internal Medicine, Nephrology Section, Faculty of Medicine, October Six University, Cairo, Egypt; 3 Department of Clinical Pathology, Stem Cells Section, Faculty of Medicine, Cairo University, Cairo, Egypt; 4 Department of Internal Medicine, Nephrology Section, Faculty of Medicine, Cairo University, Cairo, Egypt; 5 Department of Biochemistry, Faculty of Medicine, Cairo University, Cairo, Egypt

## Erratum

Following the publication of our article [1], we would like to make some corrections to the text as detailed below:

**Patients and methods**

This section includes the following text: “Due to the very small size of EVs (20-1000 nm) we had no fear of size related complications (i.e. engraftment syndrome associates MSCs transplantation) and intra-arterial EVs injection has been successfully used to reduce myocardial ischemia/reperfusion injury [18]. So, we’ve chosen the intra-arterial route to maximize EVs delivery to the kidneys.” We would like to clarify that Lai et al. [18] did not use intra-arterial injection of EVs, rather the MSC exosomes they injected reached the coronary arteries and successfully reduced myocardial IRI through arterial delivery of the MSC-EVs.

**Ethical aspects**

In this section “Amsterdam’s declarations” should read “Declaration of Helsinki”

**Declarations**

The Declaration section which reads:

“The study protocol has been approved by the health ethical committee of Sahel Teaching Hospital on April 2014. All patients have been informed verbally and a written consent for the research and publication has been signed prior any step according to Amsterdam’s declarations. No individual patient’s data were included. A written consent from both parents (for umbilical cord donors) has been obtained prior to any intervention.”

should read as follows:

“The study protocol has been approved by the health ethical committee of Sahel Teaching Hospital on April 2014. All patients have been informed verbally and written consent for participation in the research and for publication has been obtained prior to any step according to the Declaration of Helsinki. A written consent from both parents (for umbilical cord donors) has been obtained prior to any intervention.”

## References

[1] Nassar W, El-Ansary M, Sabry D, Mostafa MA, Fayad T, Kotb E, Temraz M, Saad A-N, Essa W, Adel H (2016). Umbilical cord mesenchymal stem cells derived extracellular vesicles can safely ameliorate the progression of chronic kidney diseases. Biomater Res.

